# Pairwise Interactions of Three Related *Pseudomonas* Species in Plant Roots and Inert Surfaces

**DOI:** 10.3389/fmicb.2021.666522

**Published:** 2021-07-15

**Authors:** Nesli Tovi, Tomer Orevi, Maor Grinberg, Nadav Kashtan, Yitzhak Hadar, Dror Minz

**Affiliations:** ^1^Institute of Soil, Water and Environmental Sciences, Agricultural Research Organization – Volcani Center, Rishon LeZion, Israel; ^2^Department of Plant Pathology and Microbiology, Robert H. Smith Faculty of Agriculture, Food and Environment, The Hebrew University of Jerusalem, Rehovot, Israel

**Keywords:** interactions, colonization, pairwise, co-culture, colonization patterns, root extracts

## Abstract

Bacteria are social organisms that interact extensively within and between species while responding to external stimuli from their environments. Designing synthetic microbial communities can enable efficient and beneficial microbiome implementation in many areas. However, in order to design an efficient community, one must consider the interactions between their members. Using a reductionist approach, we examined pairwise interactions of three related *Pseudomonas* species in various microenvironments including plant roots and inert surfaces. Our results show that the step between monoculture and co-culture is already very complex. Monoculture root colonization patterns demonstrate that each isolate occupied a particular location on wheat roots, such as root tip, distance from the tip, or scattered along the root. However, pairwise colonization outcomes on the root did not follow the bacterial behavior in monoculture, suggesting various interaction patterns. In addition, we show that interspecies interactions on a microscale on inert surface take part in co-culture colonization and that the interactions are affected by the presence of root extracts and depend on its source. The understanding of interrelationships on the root may contribute to future attempts to manipulate and improve bacterial colonization and to intervene with root microbiomes to construct and design effective synthetic microbial consortia.

## Introduction

One of the frontlines of synthetic biology is engineering of synthetic microbial communities (SMCs) aiming at efficient and beneficial microbiome implementation in many areas, including biosynthesis (Santoyo et al., 2012), biodegradation of complex organic matter (McCarty and Ledesma-Amaro, 2019), and improving crop quality and health (Rainey, 1999; Lugtenberg and Kamilova, 2009). Despite the fact that SMCs promise more merits than do monocultures inoculants, the utilization of such consortia is not yet widely practiced because of our limited understanding and ability to investigate and control the interactions among members of an engineered ecosystem *in situ* (Kong et al., 2018). Many studies have shown the positive effects of inoculation with a single strain of beneficial soil microbe, in particular, *Pseudomonas* species, on crop yields, health, and quality (Loper, 1988; Capper and Higgins, 1993; Weller, 2007). However, introducing monocultures into natural environments has several limitations, as they are often more sensitive to environmental changes and are not stable (McCarty and Ledesma-Amaro, 2019). Recent studies address the question of multispecies inoculum, aiming at engineering SMC to improve plant development, health, and nutrition (Kong et al., 2018; McCarty and Ledesma-Amaro, 2019; Zafar-ul-Hye et al., 2020). Zafar-ul-Hye et al. (2020) showed that multispecies inoculation improved wheat growth compared to single-strain inoculation.

In a previous study, we inoculated roots with three related *Pseudomonas* species (NT0124, NT0128, and NT0133) originated from wheat roots and followed monoculture colonization pattern on the roots (Tovi et al., 2019). Using microscopy and cultivation-based methods, we showed that all three isolates effectively colonized wheat roots and that colonization was plant species dependent. Moreover, each isolate occupied a particular location on wheat roots (i.e., root tip, scattered, or distant from the tip). However, to understand microbial community assembly and interactions between bacteria, it must be placed into an ecological context (Cordero and Datta, 2016; Stubbendieck et al., 2016).

Several studies have shown that pairwise interactions can be used to predict the composition of communities that comprised three bacterial species and may provide insight into the behavior of larger communities (Stubbendieck et al., 2016; Friedman et al., 2017; Venturelli et al., 2018; Meroz et al., 2021; Ortiz et al., 2021; Rüger et al., 2021).

In the current study, we examined whether monoculture colonization patterns of these three related *Pseudomonas* strains (Tovi et al., 2019) were sufficient to predict pairwise colonization and interaction outcomes in liquid culture, on glass surface, and in roots. Based on confocal laser scanning microscopy (CLSM), quantitative polymerase chain reaction (qPCR), and live imaging fluorescent microscopy, we demonstrated that a colonization pattern by a single isolate will not necessarily predict its behavior when introduced as a pair.

## Materials and Methods

### Bacterial Strains and Media

The bacterial strains and plasmids used in this study are listed in Table 1. The primers used are listed in Table 2. Bacteria were grown in Luria–Bertani broth (LB): 1% tryptone (Difco Laboratories Inc., United States), 0.5% yeast extract (Difco Laboratories Inc., United States), and 0.5% sodium chloride (Merck, Germany). LB was also used for bacterial soil inoculation and molecular methods (i.e., bacterial DNA extraction and plasmid purification). For the glass attachment assay and wheat or cucumber root extracts media, 50% LB media without sodium chloride was used. Solid media were prepared by adding 1.5% Bacto^TM^ agar (Difco Laboratories Inc., United States) to LB media. Where appropriate, antibiotics were added to maintain or select for plasmids as follows: for *Escherichia coli*, ampicillin (Ap, Calbiochem, United States) at 100 μg/mL and gentamicin (Gm, Sigma, United States) at 15 μg/mL and for all *Pseudomonas* isolates, Gm at 30 μg/mL.

**TABLE 1:** List of strains and plasmids used in this study and their source.

Strains	Relevant genotype or sequence	Source or references
*Pseudomonas stutzeri* NT0124	Isolated from wheat roots- PRJNA273703	Tovi et al., 2019
*Pseudomonas stutzeri* NT0128	Isolated from wheat roots- PRJNA275697	Tovi et al., 2019
*Pseudomonas fluorescens* NT0133	Isolated from wheat roots- PRJNA275699	Tovi et al., 2019
NT0124/pBT270: miniTn7T-Gm-GFP	Ap^r^ and Gm^r^, pUCP18-miniTn7T2.1 -GFP	Tovi et al., 2019
NT0128/pBT270: miniTn7T-Gm-GFP	Ap^r^ and Gm^r^, pUCP18-miniTn7T2.1 -GFP	Tovi et al., 2019
NT0133/pBT270: miniTn7T-Gm-GFP	Ap^r^ and Gm^r^, pUCP18-miniTn7T2.1 -GFP	Tovi et al., 2019
NT0128/pBT270: miniTn7T-Gm- mCherry	Ap^r^ and Gm^r^, pUCP18-miniTn7T2.1 -mCherry	This research
NT0133/pBT270: miniTn7T-Gm- mCherry	Ap^r^ and Gm^r^, pUCP18-miniTn7T2.1 - mCherry	This research
Plasmids		
pUCP18-miniTn7T2.1Gm- GFP	Ap^r^ and Gm^r^, Mini-Tn7-gfp(mut3). Integration vector for gfp.	Zhao et al., 2013
pUCP18-miniTn7T2.1Gm- mCherry	Ap^r^ and Gm^r^, Mini-Tn7-mCherry. Integration vector for mCherry.	Zhao et al., 2013
pUC18T-mini-Tn7T-Gm-eyfp	Ap^r^ and Gm^r^, Mini-Tn7-mCherry. Integration vector for yfp.	Choi and Schweizer, 2006
Ptns2	Ap^r^; helper strain for mobilizing miniTn7 into *P. aeruginosa* strains by mating	Choi and Schweizer, 2006
pGEM:tef	pGEM:tef, Ap^r^ vector for transcript elongation factor gene(tef)	Tovi et al., 2019

**TABLE 2:** List of primers used in this study and their source.

Plant *tef*_f	ACTGTGCAGTAGTACTTGGTG	Ruppel et al., 2006
Plant t*ef*_r	AAGCTAGGAGGTATTGACAAG	Ruppel et al., 2006
GFP_f	CACTGGAGTTGTCCCAATTC	Tovi et al., 2019
GFP_r	GGCCATGGAACAGGTAGTTT	Tovi et al., 2019
mCherry_f	CTACGACGCTGAGGTCAAGA	This research
mCherry_r	CGATGGTGTAGTCCTCGTTG	This research

### Molecular Methods

All basic molecular techniques were executed according to standard protocols: DNA (from roots and isolates) was extracted using soil GeneAll kit (soil DNA production kit, GeneAll, South Korea) according to the manufacturer protocol. Plasmids were purified with the QIAprep Spin Miniprep Kit (Qiagen, Germany). All primers were obtained from Integrated DNA Technologies (IDT, United States), and PCR primers are listed in Table 2. For quantifying plant gene copy number by qPCR, we used the known DNA concentration of a specific plasmid containing the target region coding for translation elongation factor 1 (*tef*). In order to examine the ability to colonize wheat roots (as described below), the isolates were chromosomally labeled with the *gfp* or *mCherry* gene. Construction of chromosomally *mCherry*-expressing strains: pUCP18-miniTn7T2.1Gm-GW: Gm-*mCherry* (Zhao et al., 2013) was inserted into *Pseudomonas* isolates (NT0128, NT0133) together with pTNS2 helper plasmid using electroporation (Choi and Schweizer, 2006; Zhao et al., 2013; Marmont et al., 2017) as described by Tovi et al. (2019). All plasmids are listed in Table 1.

### Plant Growth and Bacterial Colonization

Wheat (*Triticum turgidum* cv. Negev, Hazera, Israel) or cucumber seeds (*Cucumis sativus* cv. Kfir, Zeraim Gedera, Israel) were prepared as described previously by Tovi et al. (2019). Briefly, wheat seeds were surface sterilized by soaking in 3% sodium hypochlorite for 1.5 min, followed by 70% ethanol for 1.5 min, then rinsing three times with water. The sterilized seeds were cultivated in sterile mix of sandy loam soil with perlite 9:1 (wt/wt), hydrated with half-strength Hoagland solution (Ofek et al., 2014). For soil inoculation, bacteria were grown in LB overnight, diluted 1:100 in LB, and further grown in LB for 3–4 h and inoculated at 10^6^ bacteria per gram of soil–perlite mixture. Plants were grown for 10–12 days, after which roots were carefully removed and rinsed in sterile saline, and soil adhering to the roots was removed by vortex. Roots were weighted and used for either DNA extraction and qPCR or for imaging bacterial colonization by CLSM.

### CLSM and Sample Preparation

Roots were stained with Hoechst 3334 (NucBlue, Thermo, United States) according to the manufacturer protocol. Images were acquired using either OLYMPUS IX 81 (Olympus Corporation, Japan) inverted laser scanning confocal microscope (FLUOVIEW 500) equipped with 405-, 488-, 515-, and 543-mm laser lines and a 20 × 0.7 NA UPlanApo objective or a Leica SP8 laser scanning microscope (Leica, Germany) equipped with a solid state laser with 405-, 488-, 514-, and 552-nm light, and HC PL APO CS2 20×/0.75 objective (Leica, Germany) and Leica Application Suite X software (LASX, Leica, Germany).

### Real-Time PCR Quantification of Colonized Bacteria on Wheat Roots

Wheat roots were prepared as described above. An average of 0.25 g of wheat root was used for DNA extraction. Each sample represents three different individual roots from the same pot, and a minimum of six different replicates (individual pots) of wheat plants were used for quantification. Bacterial abundance on roots was quantified using qPCR by targeting GFP, *mCherry* (for labeled isolates), and normalizing it to the copy number of the plant *tef* gene. In all samples, GFP and *mCherry* target numbers were divided by the *tef* target number, as described by Ofek et al. (2011). A plasmid standard containing the target region was generated for the *tef* gene as described previously (Ofek et al., 2011; Tovi et al., 2019). For GFP, *mCherry*, and tef quantification, the plasmids containing the target region listed in Table 1 were used as standard, and primers used for quantifying each gene are listed in Table 2. Each gene copy number was calculated using StepOne software v2.3 (Applied Biosystems, United States), using the known DNA concentration and the specific plasmid plus the molecular weight of the insert, estimated from their lengths (Table 2). All qPCR assays were conducted in polypropylene 96-well plates and StepOnePlus real-time PCR system (Applied Biosystems, United States). Plasmids DNA concentrations were measured using Qubit fluorometric quantification (Thermo Fisher Scientific, United States) and Qubit dsDNA BR assay kit (Thermo Fisher Scientific, United States). Sevenfold dilution series of the plasmids containing the target genes were conducted within a range of 10^8^ copies per 1 mL to 10^2^ copies per 1 mL. The standards and each sample within each treatment were tested in triplicate. The slope of the standard curve, correlation coefficient, and amplification efficacy were calculated using StepOne software v2.3. Each 20-μL reaction contained 10 μL Absolute Fast SYBR^®^ Green Master Mix (Thermo, United States), 0.6 μL of each primer (100 μM), 7.8 μL H_2_O, and 1 μL template DNA (diluted 1:10). PCR conditions were as follows: 5 min at 95°C, followed by 40 cycles of 95°C for 5 s, 60°C for 30 s. Melting curve analysis of the PCR products was performed following each assay to confirm that the fluorescence signal originated from specific PCR products.

### Wheat and Cucumber Root Extracts Preparation

Wheat or cucumber roots were washed of all soil particles. The washed roots were shaken in 0.25% sodium chloride for 2 h. In order to prepare a particle free extract, the roots were centrifuged (4,500 revolutions/min, 10 min, 22°C), and the supernatant was filtered through 0.22-μm Autofill Filtration System PES (Foxx, Life Science, United States) and kept at −80°C. When needed, 50% LB without sodium chloride was added to the filtered supernatant (termed here “root extract”) for the preparation of root extract containing medium that was sterilized by filtration.

### Real-Time PCR Quantification of Isolates in Liquid Culture

*gfp*- or *mCherry*-labeled isolates were grown in LB overnight and then diluted 1:100 and left to grow up to approximately 0.4 OD in 3 mL LB. The bacteria cultures were diluted again to 1:100,000 and left to grow overnight in 50% LB or in cucumber or wheat root extract media. The overnight cultures were centrifuged, and the pellets were kept at −20°C until used for genomic DNA extraction. DNA was extracted and diluted to 20 ng/μL for each qPCR reaction. GFP or mCherry copy numbers were calculated as described above. *p* value was calculated by Mann–Whitney *U* test by comparing the monocultures (triangle) to co-cultures (circle) strains.

### Live Imaging and Kinetics of Attachment to Glass Plate Surface

*gfp*- or *mCherry*-labeled isolates were grown in LB media overnight, diluted 1:100, and left to grow up to approximately OD_600_ = 0.4 in 3 mL LB. The bacteria cultures were diluted again to a final concentration of 1:100,000 in 1 mL of 50% LB or in cucumber or wheat root extract media in glass-bottom 12-well plates (Cellvis, United States). Image acquisition was performed using an Eclipse Ti-E inverted microscope (Nikon, Japan) equipped with a Plan Apo 40x/0.95 NA air objective. An LED light source (SOLA SE II, United States) was used for fluorescence excitation. GFP fluorescence was excited with a 470/40 filter, and emission was collected with a T495lpxr dichroic mirror and a 525/50 filter. *mCherry* fluorescence was excited with a 560/40 filter, and emission was collected with a T585lpxr dichroic mirror and a 630/75 filter (all filters from Chroma Technology Corp., Brattleboro, VT, United States). Images were acquired with an SCMOS camera (ZYLA 4.2 PLUS, Andor, United Kingdom) at 1-h intervals for a period of 17 h. NIS software (version 5.02, Nikon Instruments, Inc.) was used for acquisition and basic image processing.

### Spatial Analysis for Pairwise Interaction of Two Isolates on a Glass Surface

Pair cross-correlation (PCC) function *g*(*r*) (Lotwick and Silverman, 1982) describes the spatial organization of two strains on a glass surface at several time points (Figure 4). In general, PCC quantifies the relationship between a pair of populations by examining the distances between their respective members. For a given pair of populations on a two-dimensional (2D) surface,

(1) g(r)=p(r)/2DD12

where *p*(*r*) is the probability of finding a pair of members of both populations at distance *r*, and *D*_*i*_ is the fraction of area covered by population *i*. The mean and confidence interval of *g*(*r*) can be calculated by combining the analysis results of several separate 2D areas (Steinberg et al., 2021). If the confidence interval of *g*(*r*) at distance *r* includes 1, then the two populations do not exhibit significant attraction or repulsion with respect to each other at distance *r*; if *g*(*r*) at distance *r* is significantly higher than 1, then the populations are clustered at distance *r*, and conversely, if *g*(*r*) at distance *r* is significantly lower than 1, then the populations are repelled at distance *r*. PCC analysis was performed by the 2D dipole algorithm, using DAIME software package (Daims et al., 2006). Mean and 95% confidence intervals were calculated by pooling the results of 2 × 2 sections of each image (total image area is 0.85 mm^2^ at 0.16 μm/pixels). The analysis is performed at the pixel level; therefore, the measurements pertain to a continuous fraction of the population by area. The analysis parameters are a sample size of 500,000 dipoles and binning of distances at 0.96-μm intervals.

### Statistical Analysis

Statistical analysis was performed using the Mann–Whitney *U* test, and *p* < 0.01 was considered statistically significant.

## Results

### Spatial Scales of Interactions and Abundance of Three Pairs of Related *Pseudomonas* Species on Wheat Roots

**FIGURE 1:**
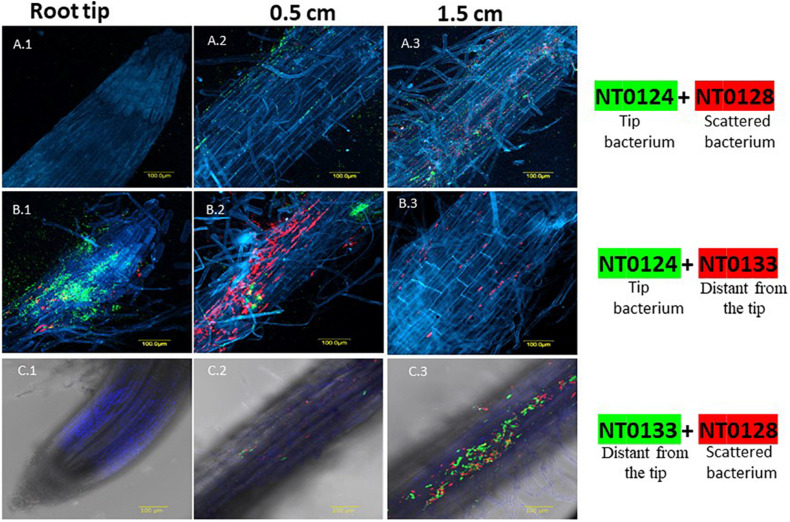
Spatial distribution affected by co-colonization of *Pseudomonas* species pairs on wheat roots evaluated by CLSM. Wheat roots were sampled after 10–12 days of growth in soil inoculated with GFP and mCherry labeled isolates: NT0124 (green) and NT0128 (red) **(A.1–A.3)**; the pair NT0124 (green) NT0133 (red) **(B.1–B.3)**; the pair NT0128 (red) and NT0133 (green) **(C.1–C.3)**. Root is labeled in blue using Hoechst dye. In all panels, the labeling is as follows: **(1)** root tip, **(2)** 0.5 cm from the root tip, **(3)** 1.5 cm from root tip. The various sections of each pair were taken from the same root. The images represent multiple roots taken from at least two different individual experiments. For spatial distribution of roots colonization by the monocultures see Tovi et al. (2019); Figure 4A.

**FIGURE 2:**
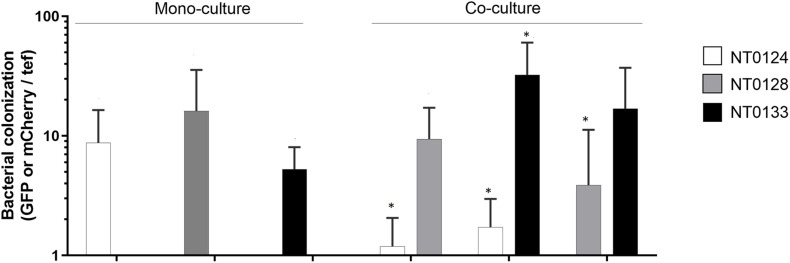
Bacterial abundance is affected by pairwise interaction on wheat root. Wheat root colonization by monoculture and pairs of isolates on 10- to 12-day-old whole-root samples of wheat. Real-time qPCR analysis was conducted by quantifying *gfp* and *mCherry* copy numbers and normalizing to plant *tef* copies. Error bars indicate standard deviation (*n* > 6). Bacterial root colonization significances were measured by comparing the difference between the isolate abundance in monoculture to its abundance in co-culture. Colonization of each species in co-cultures, which differ significantly from its monoculture are labeled with an asterisk (*). Mann–Whitney *U* test analysis revealed significant difference for strains: NT0124 + NT0128 (NT0124, *p* = 0.005; NT0128, *p* = 0.43 *n* = 6); NT0124 + NT0133 (NT0124, *p* = 0.006; NT0133 *p* = 0.0006; *n* = 7) and NT0128 + NT0133 (NT0128, *p* = 0.01; NT0133, *p* = 0.29; *n* = 8).

To assess whether it is possible to predict co-cultures outcomes from monocultures behavior, we followed and examined spatial scales of interactions and abundance of three pairs of related *Pseudomonas* species. These strains have previously been shown to colonize roots differently when inoculated separately: NT0124 (tip colonizer) is specialized in efficient colonization of the root tips; NT0128 colonizes along the root; and NT0133 colonizes distant from the tip (Tovi et al., 2019). In the current study, we followed bacteria inoculation in pairs using two methods: qualitatively on root topography, using CLSM (Figure 1), and quantitatively, using qPCR analysis (Figure 2).

Wheat seedlings were grown in soil inoculated with the various pairs. The pattern of root colonization was followed in 10- to 12-day-old seedlings using CLSM. Remarkably, CLSM images of each pair that were taken from the coinoculated roots showed that the spatial distribution of each strain could be either affected or independent of the companion strain (Figure 1). Co-inoculation of isolates NT0124, the “tip bacterium” (shown in green) and NT0128 (in red) exhibited a root-wide colonization distribution: Both isolates colonized along the root, without apparent preference for a specific area (Figures 1A.1–A.3). When inoculated together with NT0128, the spatial distribution preference of the “tip bacterium” (NT0124) changed, and it was absent from the tip (Figure 1A.1). In contrast, Co-inoculations of NT0124 with NT0133 retained their original spatial preference, with each isolate occupying its preferred specific niche on the root (Figures 1B.1–B.3), as was the case when inoculated as single culture: NT0124 (shown in green) was localized mainly at the root tip (Figure 1B.1), and NT0133 (in red) was localized at the distant part (Figure 1B.2). Similarly, the pair NT0128 and NT0133 did not change its typical spatial distribution on wheat roots (Figures 1C.1–C.3): NT0128 (in red) with NT0133 (in green) colonized the same region: the zone distant from the tip (Figure 1C.3).

Bacterial abundance on the inoculated wheat roots was quantified by examining each strain separately and in pairs, using qPCR by targeting *gfp* or *mCherry* genes of each isolate. The plant gene *tef* (translation elongation factor), representing plant cell numbers, was quantified for normalization (Figure 2). Interestingly, qPCR results showed that even though the spatial distribution pattern of the pairs NT0124 with NT0133 and NT0128 with NT0133 did not change (Figure 1A), their abundance was affected significantly: NT0124 abundance on the roots decreased when inoculated with NT0133 (*p* = 0.006, Mann–Whitney *U* test, *n* = 7), whereas NT0133 abundance increased (*p* = 0.0006, Mann–Whitney *U* test, *n* = 7), compared to plants that were inoculated with each of these strains separately. Similarly, when NT0128 was inoculated on the roots together with NT0133, NT0128 abundance significantly decreased (*p* = 0.01, Mann–Whitney *U* test, *n* = 8), whereas NT0133 abundance was similar to its abundance as a monoculture. When NT0124 was coinoculated with NT0128, the abundance of NT0124 decreased (*p* = 0.005, Mann–Whitney *U* test, *n* = 6), whereas NT0128 abundance on the roots was not affected (Figure 2).

### Monoculture and Co-culture Colonization Dynamics on Glass Surfaces

**FIGURE 3:**
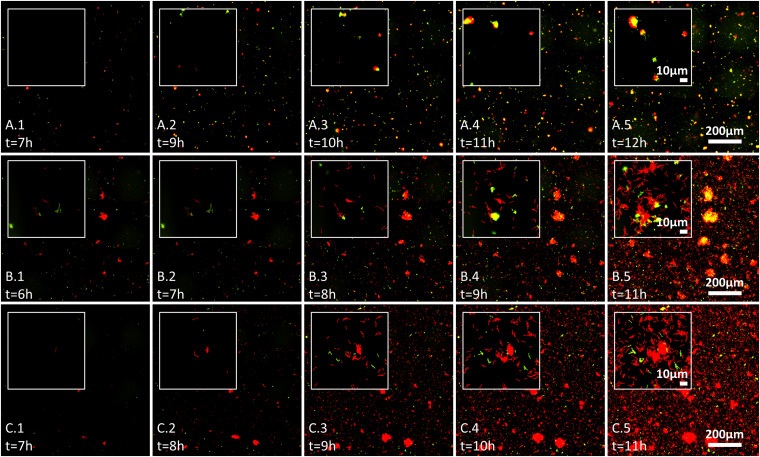
Effect of wheat root extracts on bacteria–bacteria interactions on glass surface, evaluated by live imaging microscopy. Bacterial surface colonization was evaluated over 17 h. Images from 6 to 12 h are shown, and time of measurement is indicated in each image. Each image represents the whole analyzed field of view (scale 200 μm) with inset showing the surface at scale of ∼10 μm. Labeled GFP and mCherry bacteria are shown in red and green (yellow represents red and green overlay) and the labeling is as follows: **(A.1–A.5)** pair NT0124 (green) and NT0128 (red); **(B.1–B.5)** pair NT0124 (green) and NT0133 (red), and **(C.1–C.5)** pair NT0128 (green) and NT0133 (red). The images represent results of at least two independent experiments.

**FIGURE 4:**
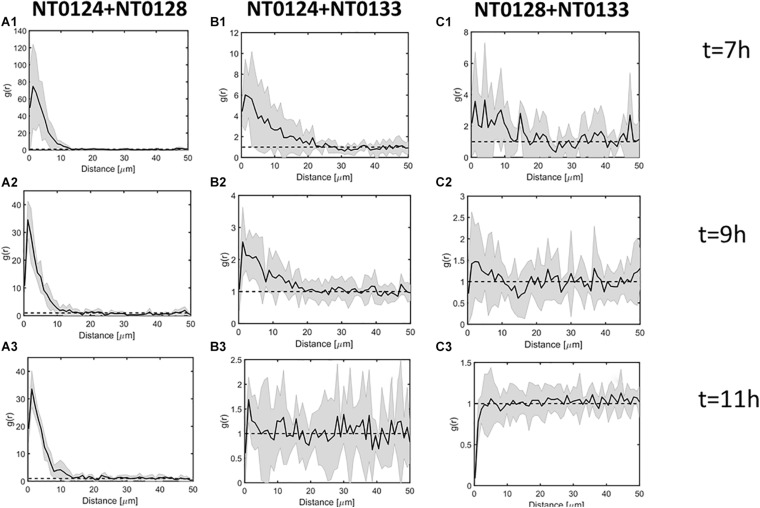
Spatial analysis of pairwise interactions between isolates during surface colonization process. Pair cross-correlation function *g*(*r*) at *t* = 7, 9, 11 h. Black line and gray envelope represent the mean and 95% confidence interval, respectively (see section “Materials and Methods”). In all panels, the label is as follows: (1) Images at time 7 h; (2) images at time 9 h; (3) images at time 11 h; **(A)** pair NT0124 and NT0128, exhibiting the strongest co-localization, reflected by *r* > 1 at distances <∼10 μm; **(B)** pair NT0124 and NT0133 exhibiting intermediate co-localization, reflected by *r* > 1 at distances <5 μm; **(C)** pair NT0128 and NT0133 showing no significant co-localization (*r* does not deviate from 1 at all distances).

In order to rule out the effect of the physical and chemical properties of the plant roots on bacterial interactions and colonization, we studied monocultural and cocultural behavior on an inert surface (glass) when treated with either LB or root extracts. Bacterial surface colonization and attachment were examined by live imaging microscopy (Figures 3, 4). Pairwise interactions were also examined in liquid cultures, an environment wherein attachment to the surface is eliminated (see below). Live imaging was enabled following the dynamics of surface colonization, including attachment and aggregate formation on the glass surface, while monitoring the interactions over time. On the inert surface, in the presence of nutrients, the attachment of both monocultures, NT0124 and NT0128, was limited (Supplementary Figures 1A.1,A.2) (Supplementary Figures 1B.1,B.2). Under the same conditions, NT0133 was able to attach to the glass surface and to form aggregates (Supplementary Figure 1A.3). We also examined the ability of the three isolates to attach to glass in response to wheat or cucumber root extract (Supplementary Figures 1B.1–B.3,C.1–C.3). In the presence of wheat root extract, isolates NT0124 and NT0128 attached to the glass surface and formed micro-colonies (Supplementary Figures 1B.1,B.2, respectively) in a similar manner to that of NT0133 (Supplementary Figure 1B.3). In contrast, none of the isolates changed their attachment patterns to the surface in response to cucumber root extract (Supplementary Figures 1C.1,C.2).

We further followed bacterial colonization and aggregate formation dynamics on glass surface in the three pairs over time in the presence of wheat root extract. In the pair NT0124 and NT0128 (green and red, respectively; Figures 3A.1–A.5), both strains colonized the glass surface. Moreover, cells of isolate NT0124 appeared to attach to NT0128 aggregates from very early stages of their colonization, along all time points, regardless of aggregate size (Figures 3A.1–A.5). The pair NT0124 and NT0133 (green and red, respectively; Figures 3B.1–B.5) exhibited a differing pattern: NT0124 cells attached to NT0133 aggregates only after the latter grew bigger over time up until NT0133 covered the surface. In the pair NT0128 and NT0133 (green and red, respectively; Figures 3C.1–C.5), both colonized the glass surface and formed microcolonies without any observed interspecies preference.

To assess whether the spatial organization generated by each pair during the colonization process is indicative of interaction between the isolates, the PCC function *g*(*r*) was used (Figures 4A–C; see section “Materials and Methods” for more details). If *g*(*r*) at distance *r* is significantly higher than 1, then the two strains were colocalized at distance *r* more than expected by chance. If *g*(*r*) is significantly lower than 1, the two strains repel each other at distance *r*. Otherwise, if *g*(*r*) does not significantly deviate from 1, then the interaction cannot be distinguished.

The pair NT0124 and NT0128 exhibited the strongest co-localization at distances under 10 μm (Figure 4A.1). The pair NT0124 and NT0133 exhibited intermediate co-localization at distance of 5 μm (Figure 4B.1) up until NT0133 covered the surface (Figure 4B.3). In contrast, the organization of the pair NT0128 and NT0133 did not deviate from the expected by random at any range; therefore, no significant co-localization was observed. Thus, NT0128 and NT0133 spatially dependent interaction on the glass surface can be assumed to be weak (Figure 4C.1). In all three pairs, similar results were observed for later time points (Figure 4, panels 2–3).

### Bacterial Interactions in a Liquid Medium

**FIGURE 5:**
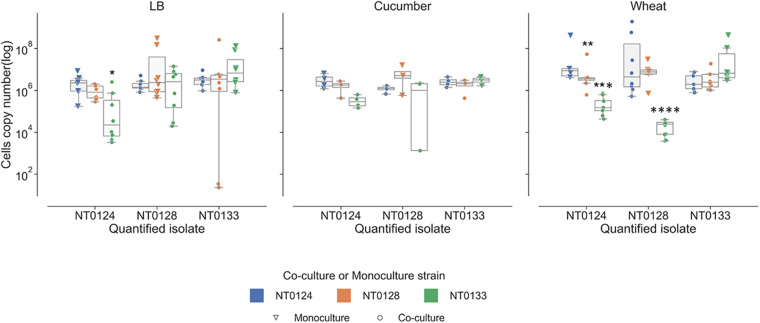
Wheat root extracts affect bacterial interactions in a liquid medium. Real-time qPCR analysis was performed by quantifying copy numbers GFP or mCherry of monocultures (triangle) or co-cultures (circle) strains in 50% LB, wheat root extract media, and cucumber root extract media. The colors indicate the partner strain: NT0124 blue; NT0128 orange; NT0133 green. *Copy numbers values are significantly different (*p* < 0.02) in the co-culture compared to the corresponding monoculture. *p* value was calculated by Mann–Whitney *U* test and represents at least four different independent samples that were used for quantification.

Bacterial interactions were examined in liquid containing LB, wheat root extracts, or cucumber root extracts. Under these conditions, interactions are not related to surface attachment as they grow in planktonic mode rather than forming a biofilm. The abundance of each of the isolates in monoculture and in pairs was measured using qPCR (Figure 5). In LB cultures, population levels of all three pairs (NT0124 with NT0128, NT0124 with NT0133, and NT0128 with NT0133) were similar to their levels as monocultures (Figure 5, left-hand subplot). However, when grown in the presence of wheat root extract (Figure 5, right-hand subplot), in pairs, different interactions were observed. In a culture of NT0124 with NT0133, isolate NT0124 abundance decreased significantly (*p* = 0.001, Mann–Whitney *U* test, *n* = 9) compared to its growth alone. This interaction could be considered amensalism. On the roots, the interaction between these two strains may be considered antagonistic, as the population of NT0133 increased. Likewise, in co-culture NT0128 with NT0133, the abundance of NT0128 decreased significantly (*p* = 0.001, Mann–Whitney *U* test, *n* = 10). However, co-culture NT0124 with NT0128 may be explained as neutralism; that is, the growth of each isolate was unaffected by the other (*p* = 0.8, Mann–Whitney *U* test, *n* = 8). In a medium containing cucumber root extracts, the growth of all three strains in pairs was similar to their growth as monocultures (Figure 5, middle subplot).

## Discussion

Interspecies interactions affect the establishment and success of beneficial bacterial consortia (Rajput et al., 2018; Gao et al., 2020). Here we took a reductionist approach to study pairwise bacterial interactions on root and inert surfaces. In a recent review, Romano et al. (2020) concluded that for successful root colonization, tracking and monitoring of inoculated microorganisms should employ a variety of methodological approaches (Romano et al., 2020). In the current study, we employed several, complementary methods to examine pairwise interspecies interactions, culturing, CLSM, qPCR, and live imaging microscopy. Here, the interactions between closely related *Pseudomonas* species previously studied individually (Tovi et al., 2019) were investigated. We focused on dual-species interactions during early phases of colonization of wheat roots as well as on inert surface (glass).

Producing effective bacterial inoculant requires the ability of successful colonization and adherence to plant roots surfaces, establishing a compatible interaction within the community, and persistence via competence traits, such as utilization of the plant deposits and biofilm formation (Finkel et al., 2017; Herrera Paredes et al., 2018; Santhanam et al., 2019; Romano et al., 2020). For example, Santhanam et al. (2019) described three members of a bacterial consortium designed to protect plants from wilting: *Pseudomonas azotoformans*, *Pseudomonas frederiksbergensis*, and *Arthrobacter nitroguajacolicus*, each of which forms biofilms when grown individually, but the quantity of biofilm improved synergistically in a five-member consortium (Santhanam et al., 2019).

In the current study, the mutual behavior of the isolates, all originated from wheat roots, was influenced by wheat root extract but not by cucumber or LB medium. Importantly, our experiments, either on roots or on inert surfaces, demonstrate that bacteria perform differently in monocultures than in co-cultures. This difference is manifested, for example, by the absence of the “tip bacterium” (NT0124) from the root tip when coinoculated with NT0128, a non-specialized colonizer. Together, both isolates still colonized the root efficiently, but without apparent preference for location. On an inert surface, strain NT0124 attached to aggregates formed by NT0128 only in response to wheat root extract, but not to cucumber root extracts, suggesting that the plant host plays a specific role in modulating bacterial cell–cell interactions and colonization behavior. On the other hand, the “tip bacterium” population size was reduced in the presence of NT0133, suggesting possible competition. This might be despite the inferior motility traits of NT0133 (Tovi et al., 2019); it was previously suggested that superior motility promotes competitive exclusion (Hibbing et al., 2010; Wiles et al., 2016). Thus, motile bacteria could have an advantage in competition during root colonization (De Weert et al., 2004). Consequently, two species that compete for the exact same resources have several options: one strain can exclude or dominate the other; they can both coexist or move toward differing niches, thus utilizing differing resources. Here, the less motile bacterium (NT0133) was able to outcompete the more motile isolate (NT0124). On the inert surface, the interaction differed: NT0124 was attracted to microcolonies of NT0133 as these microcolonies grew bigger. On the roots, however, in the presence of NT0124, the NT0133 aggregates were smaller than on the inert surface, possibly suggesting that competition with NT0124 on the roots resulted in smaller NT0133 aggregates. When NT0133 was inoculated together with NT0128 on the roots, the colonization pattern of NT128 suggested antagonism. However, such antagonism was not observed between NT0133 and NT0128 when colonizing the inert surface. Indeed, the root environment differs physically, chemically, and microbially from the glass surface.

In the current work, by studying dual-species interactions in various “habitats” roots, inert surfaces, and liquid media, we showed that the outcome of such interactions is strongly affected by environmental conditions. Our results indicate that studying individual strain inoculant is not sufficient for selecting the best colonizer or for predicting its success in a consortium. In addition, bacterial traits such as motility and aggregate formation, known to be important for spatial colonization of a single isolate, will not necessarily determine its success within a multispecies consortium. Bacteria are social organisms that interact extensively within and between species, while responding to external stimuli from their environments, thus leading to selection for variants that are better equipped to colonize various niches. Bacterial interaction and the specific niche should be considered in future attempts to manipulate microbiomes by introducing beneficial bacteria and, in particular, in constructing SMCs (Satyanarayana et al., 2019; Gao et al., 2020).

## Data Availability Statement

The raw data supporting the conclusions of this article will be made available by the authors, without undue reservation.

## Author Contributions

NT, YH, NK, and DM designed the experiments. NT preformed the experiments. NT and TO preformed the live imaging and kinetics of attachment. MG analyzed bacterial spatial analysis on glass surface. All authors jointly wrote the manuscript.

## Conflict of Interest

The authors declare that the research was conducted in the absence of any commercial or financial relationships that could be construed as a potential conflict of interest.

## Supplementary Material

Supplementary Figure 1 Surface colonization dynamics of *Pseudomonas* species cultures on a glass surface, evaluated by live imaging microscopy. Colonization dynamics of GFP-labeled isolate was evaluated over 17 h; images at 10 or 11 h are shown **(A–C)**. **(A.1–A.3)** Medium with no root extracts, **(B.1–B.3)** medium supplemented with wheat root extracts, and **(C.1–C.3)** medium supplemented with cucumber root extracts. The images show results of at least two independent experiments.
