# Supplementary figures and images for: Pairwise Interactions of Three Related Pseudomonas Species in Plant Roots and Inert Surfaces
Source: Front Microbiol. 2021 Jul 15;12:666522. doi: 10.3389/fmicb.2021.666522 (PMC8320352; doi:10.3389/fmicb.2021.666522)

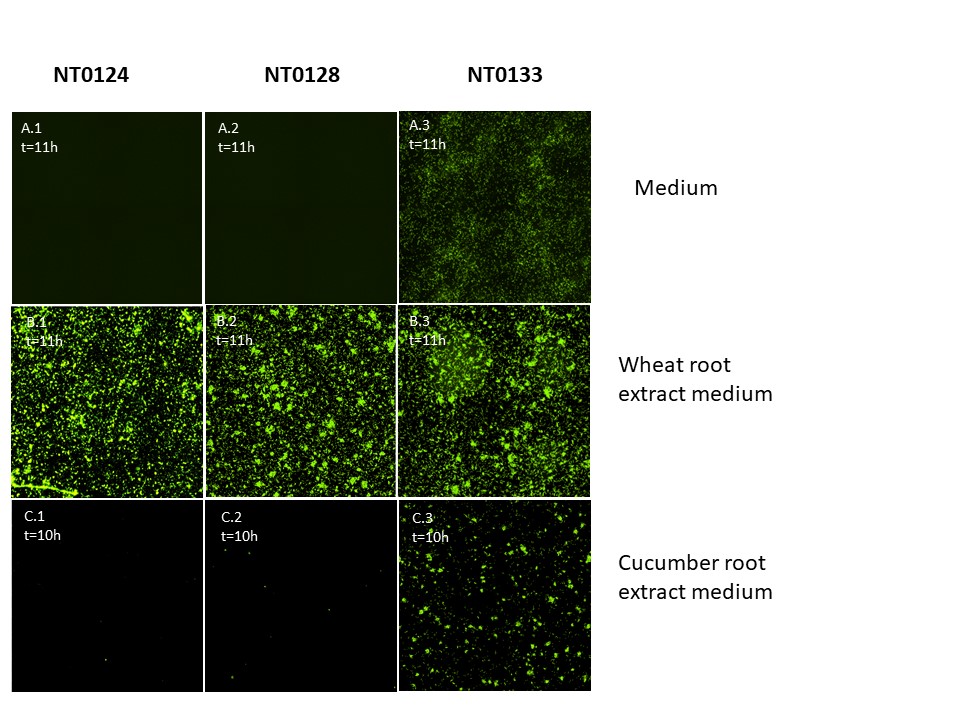

Supplement: Supplementary Figure 1 — Surface colonization dynamics of Pseudomonas species cultures on a glass surface, evaluated by live imaging microscopy. Colonization dynamics of GFP-labeled isolate was evaluated over 17 h; images at 10 or 11 h are shown (A–C). (A.1–A.3) Medium with no root extracts, (B.1–B.3) medium supplemented with wheat root extracts, and (C.1–C.3) medium supplemented with cucumber root extracts. The images show results of at least two independent experiments. [file Image_1.JPEG]
